# Tonnis Angle and Acetabular Retroversion Measurements in Asymptomatic Hips Are Predictive of Future Hip Pain: A Retrospective, Prognostic Clinical Study

**DOI:** 10.5435/JAAOSGlobal-D-20-00213

**Published:** 2020-12-07

**Authors:** Alfred Mansour, Patrick M. Carry, Matthew Belton, Kaley S. Holmes, Christopher J. Brazell, Gaia Georgopoulos, Bryant Elrick, Ernest Sink, Nancy Hadley Miller

**Affiliations:** From the Department of Orthopaedics, McGovern Medical School at UTHealth, Houston, TX (Dr. Mansour); Musculoskeletal Research Center, Children's Hospital Colorado, Aurora, CO (Mr. Carry, Ms. Holmes, and Mr. Brazell); the Department of Orthopedics, University of Colorado Denver Anschutz Medical Campus (Dr. Belton, Dr. Georgopoulos, and Dr. Miller); University of Colorado School of Medicine, Anschutz Medical Campus (Mr. Elrick); Hospital for Special Surgery, New York, NY (Dr. Sink); and Weill Cornell Medical School (Dr. Sink).

## Abstract

**Background:**

This study evaluated the prevalence of radiographic abnormalities potentially indicative of femoroacetabular impingement on AP pelvic radiographs in asymptomatic adolescents and young adults and aimed to determine whether the abnormalities were predictive of future hip pain.

**Methods:**

AP pelvis images from scoliosis radiographs were obtained from patients 12 to 25 years of age free of any clinical hip/lower extremity symptoms between January 2006 and September 2009. The following radiographic abnormalities were collected: lateral center-edge angle of Wiberg >40° or <25°, Tönnis angle <0° or >10°, acetabular retroversion (crossover sign with a posterior wall sign), acetabular overcoverage (crossover sign without a posterior wall sign), and anterior offset alpha angle, calculated using alpha angle of Nötzli >50°. Patients were retrospectively followed (average 3.11 years) to identify those who subsequently developed hip pain.

**Results:**

Of the 233 patients (466 hips) who were asymptomatic at the time of radiographic evaluation, at least one radiographic abnormality was present in 60% (281/466) of the hips. Within that group of hips (n = 281), 69% (195/281) of hips demonstrated a single abnormality, whereas 31% (86/281) of hips were associated with multiple abnormalities. Among all hips (n = 466), a lateral center-edge angle <25° or >40° was the most common radiographic abnormality, present in 27% (127/466) of hips. Anterior offset alpha angle and acetabular overcoverage were the most common abnormalities to present together, found in 5% (25/466) of hips. In the multivariable model, a decreasing Tönnis angle (hazard ratio per 1-degree decrease: 1.25, 95% confidence interval, 1.10–1.42, *P* = 0.0006) and the presence of acetabular retroversion (hazard ratio: 3.55, 95% confidence interval, 1.15–10.95, *P* = 0.0272) were predictive of the development of future hip pain.

**Conclusions:**

Our study demonstrates a high prevalence of radiographic abnormalities indicative of femoroacetabular impingement in asymptomatic adolescents and young adults. A decrease in Tönnis angle and the presence of acetabular retroversion were predictive of future hip pain.

Femoroacetabular impingement (FAI) is a pathological mechanical process within the hip joint resulting from either acquired or developmental hip morphology. It is a common source of hip pain in young and active patients, with a prevalence between 10% and 15%.^1,2^ The following three forms of FAI exist: pincer impingement, which is characterized by overgrowth of the acetabulum, cam impingement, which is characterized by abnormal sphericity of the femoral head, and a mixture of both types of FAI, which can lead to limited motion, joint damage, pain, and early osteoarthritis.^1,2,3,4,5,6^ FAI has been noted as a factor related to early onset degenerative osteoarthritis.^3,5^ Early diagnosis of FAI has been suggested as potentially important because it may allow for early intervention to correct biomechanical factors that are related to irreversible chondral damage.^1,2^

Radiographic evidence of FAI can assist in clinical decision-making, but multiple reports have highlighted asymptomatic populations with radiographic evidence of FAI.^7,8^ A systematic review evaluating 2,114 asymptomatic hips found that 67% had radiographic evidence of a pincer deformity, and 37% had evidence of a cam deformity.^9^ This review by Frank et al, along with other reviews, mainly focused on adult populations.^1^ Only a handful of reports have evaluated radiographic evidence of FAI in asymptomatic adolescent populations.^10-12^ More importantly, it is assumed that the radiographic abnormalities precede the onset of hip pain. The clinical dilemma is the determination of those specific individuals whose radiographs have parameters indicating FAI and will develop early onset degeneration. No previous study has followed asymptomatic adolescent patients to determine whether the radiographic abnormalities, present at the baseline asymptomatic visit, increase the risk of future hip pain with subsequent cartilage degeneration.

The aim of this cross-sectional study was to establish the prevalence of radiographic abnormalities indicative of FAI among asymptomatic adolescents and young adults. Using a cohort study design, we also aimed to determine whether the radiographic abnormalities obtained from the cross-sectional portion of the study predicted the development of future hip pain.

## Methods

After institutional review board approval, we retrospectively reviewed the pelvic portion of standing spinal radiographs obtained from individuals referred for potential idiopathic scoliosis (IS) at a single quaternary-referral children's hospital from January 2006 through September 2009. We included all radiographs obtained from patients 12 to 25 years of age with no previous hip complaints, no known hip deformity, or diagnosis of a neuromuscular disorder. Patients with pelvic radiographs with open triradiate physes were excluded.

Radiographs were independently reviewed by a fellowship-trained pediatric orthopaedic surgeon with experience in hip preservation. The AP pelvis portions of the images were analyzed to confirm that the pelvis was centered and lacked any significant rotation in all planes (including flexion/extension). Lack of rotation was confirmed through correct alignment of the sacrum with the pubic symphysis in the coronal plane and a distance of 1 to 4.5 cm measured between the sacrococcygeal joint to the superior border of the pubic symphysis.^2^

Images were reviewed using (PACSWEB) the digital measurement software tools to evaluate the following five radiographic abnormalities described as potential indicators of FAI: lateral center-edge (LCE) angle of Wiberg >40° or <25°, Tönnis angle < 0° or >10°, acetabular retroversion (crossover sign with a posterior wall sign), acetabular overcoverage (crossover sign without a posterior wall sign), and anterior offset alpha angle (AOAA) (calculated using alpha angle of Nötzli >50°).^13^

A cohort study design was used to determine whether patients in the cross-sectional analysis developed hip pain after the baseline radiographic evaluation. Only patients with one or more clinical follow-up visits were included in the cohort analysis. Patient charts were retrospectively reviewed to identify the incidence of hip pain, defined as a report of pain in the groin during routine daily activity that may or may not increase with higher-intensity activities such as sports. Patients were followed for an average of 3.1 years (±2.8, range, 0.1 to 11.8 years) after their baseline radiograph.

Descriptive statistics were used to estimate the prevalence of the five hip radiographic abnormalities according to the number of hips included in the data set (hip level prevalence) and the number of patients included in the data set (patient level prevalence). In the cohort analysis, Cox-proportional hazard models were used to test the association between radiographic abnormalities at baseline and the incidence of hip pain. Correlation because of the inclusion of multiple hips per patient was accounted for in the statistical models. All radiographic abnormalities were tested in separate models. Radiographic variables significantly associated with onset of hip pain were tested together in the final, multivariable model. Age and sex were included as covariates in all statistical models.

## Results

A total of 233 patients (466 hips) met the inclusion criteria. Consistent with the demographics among patients referred for treatment of adolescent IS at our institution, 78% were women (181/233) and 22% were men (52/233). The mean age at the baseline radiograph was 14.7 years (±2.0, range, 11 to 20 years old). The average LCE angle was 31.9° (range, 16.0 to 51.0), and the average Tönnis angle was 4.8° (range, −12.0 to 18.7°) among all hips.

### Prevalence Among Hips

Most hips included in the final analysis, 60% (281/466), presented with at least one and as many as four radiographic abnormalities (Table 1). Among hips with one or more radiographic abnormalities (n = 281, Table 1), 31%, 86/281, of the hips were associated multiple radiographic abnormalities, whereas the remaining 69% (195/281) were associated with a single abnormality.

**Table 1:** Hip and Patient Level Prevalence

Factor	Hip Level Prevalence (n = 466), n (%)	Patient Level Prevalence (n = 233), n (%)
Parameter		
LCE angle <25° or >40°	127 (27)	95 (41)
Tönnis <0° or >10°	43 (9)	31 (13)
Acetabular retroversion	97 (21)	61 (16)
Acetabular overcoverage	61 (13)	46 (20)
Anterior offset alpha angle	66 (14)	37 (16)
No. of parameters		
0	185 (40)	65 (28)
1	195 (42)	99 (42)
2	62 (13)	44 (19)
3	21 (5)	17 (7)
4	3 (1)	8 (3)

LCE = lateral center edge

An LCE angle <25° or >40° was the most common abnormality, prevalent in 27% (127/466) of the hips (Table 1). A Tönnis angle <0° or >10° was the least common FAI abnormality, prevalent in only 9% (43/466) of the hips. The radiographic abnormalities also tended to present in patterns (Table 2). An LCE <25° or >40° was the most commonly isolated abnormality.

**Table 2:** Patient Level Prevalence Patterns

Abnormality	Absent, n (%)	Present in One Hip Only, n (%)	Present in Both Hips, n (%)
LCE angle <25° or >40°	138 (59)	63 (27)	32 (14)
Tönnis angle <0° or >10°	202 (87)	19 (8)	12 (5)
Acetabular retroversion	172 (74)	25 (11)	36 (15)
Acetabular overcoverage	187 (80)	31 (13)	15 (6)
Anterior offset alpha angle	196 (84)	8 (3)	29 (12)

LCE = lateral center-edge

We also estimated the most frequent radiographic abnormality pairings. Acetabular overcoverage was most likely to present in combination with a AOAA (5%, 25/466); acetabular retroversion was most likely to present with AOAA (4%, 20/466), and a Tönnis angle <0° or >10° was most likely to present with an LCE angle <25° or >40° (3%, 16/466).

### Prevalence Among Patients

At the patient level, one or more radiographic abnormalities were present in at least one hip in 72% of the patients (Table 1). The abnormalities tended to be bilateral in nature, indicating a high level of correlation between hips (Table 2).

### Incidence of Future Hip Pain

The hips were followed for an average of 3.1 years after the initial baseline, radiograph. Among these hips, 13 (3%) developed hip pain. The median time to onset of hip pain was 3.3 years (range, 2.1 to 6.7 years). We tested the association between radiographic abnormalities at baseline and the hazard of hip pain (Table 3). After adjusting for age at radiograph and sex, a decreasing Tönnis angle (hazard ratio per 1-degree decrease: 1.25, 95% confidence interval, 1.10–1.41, *P* = 0.006) and presence of acetabular retroversion (hazard ratio: 3.55, 95% confidence interval, 1.15–10.95, *P* = 0.0272) were the only radiographic abnormalities significantly associated with the incidence of hip pain.

**Table 3:** Hazard of Hip Pain

Parameter	HR	LCL	UCL	*P*
Tönnis angle: per 1° decrease	**1.23**	1.06	1.45	**0.0047**
Tönnis angle: abnormal vs normal^a^	**4.95**	1.09	22.41	**0.0378**
Acetabular retroversion	**2.99**	1.01	8.86	**0.0476**
No. of (+) signs	1.62	0.93	2.83	0.0906
LCE angle: per 1° increase	1.06	0.95	1.17	0.2999
Anterior offset alpha angle	0.50	0.06	3.95	0.5078
LCE angle: abnormal vs normal^b^	1.32	0.40	4.38	0.6512
Acetabular overcoverage	1.17	0.32	4.24	0.8106

HR = hazard ratio, LCE = lateral center edge, LCL = lower control limit, UCL = upper control limit

a Abnormal >10° or <0°.

b Abnormal <25° or >40°. The p-values listed indicate the significance of these statistics.

## Discussion

In a large cohort of 233 adolescent individuals without hip symptomatology, 60% of hips had at least one radiographic abnormality indicative of FAI. Among these hips, 69% of hips were associated with a single abnormality, whereas 31% of hips were associated with multiple radiographic abnormalities. Midterm follow-up revealed that a decreased Tönnis angle and the presence of acetabular retroversion were predictive of future hip pain.

Our study represents a large cohort of initially asymptomatic adolescent and young adult individuals with standard pelvis radiographs (466 hips). Schmitz et al^11^ evaluated hip morphology (cam, crossover, coxa profunda, ischial spine sign, lateral center edge, and alpha angle) in 90 asymptomatic patients (180 hips) using an EOS Imaging technique.^11^ Coxa profunda was present in 81.7% of the hips. Nepple et al^14^ have discouraged the use of coxa profunda to diagnose pincer impingement because of its high prevalence in asymptomatic hips. Based on these factors, we did not include coxa profunda in the current study. Schmitz et al^11^ also observed that acetabular overcoverage (as measured by the lateral center edge angle) was present in 15% of asymptomatic hips. Acetabular overcoverage, defined in our study as the presence of the crossover sign without a posterior wall sign, was present in 13% of the hips in our study. Acetabular over or under coverage, LCE <25° or >40°, was present in 27% of the hips in our study. The current study corroborates the high prevalence of radiographic FAI abnormalities among asymptomatic individuals.

Our results also demonstrate a high level of within patient correlation, as indicated by the high prevalence of radiographic abnormalities present bilaterally (Table 2). Correlation between affected and unaffected hips, as represented by bilateral presentation of FAI signs, has also been noted in related studies.^8,14^ Specific radiographic abnormalities also tended to be correlated. Acetabular overcoverage and an AOAA were the most prevalent combination of abnormalities, present in 5% of hips.

A major limitation of the previous cross-sectional designs used to estimate the prevalence of FAI abnormalities in asymptomatic hips has been the lack of follow-up data to determine whether abnormalities are predictive of future hip pain. Assuming that anatomic variants contribute to future hip pain, the anatomic abnormalities must precede the onset of hip pain. FAI abnormalities could represent latent hip pain. Therefore, we followed patients to determine whether patients sought care for hip pain after the baseline radiograph. In the final multivariable analysis, after adjusting for age and sex, a decreasing Tönnis angle and the presence of acetabular retroversion were significantly associated with the onset of hip pain. Although not significant, hips that developed pain were associated with a higher number of FAI abnormalities, 1.2, compared with hips that did not develop pain, 0.8. These findings suggest that FAI radiographic abnormalities may have utility in the overall clinical and radiographic assessment and prediction of future hip pain.

This study includes several limitations. The cross-sectional analysis was based on AP spinal radiographs among individuals referred for potential IS, which may not represent a true cross-section of asymptomatic subjects. In addition, the review of radiographic imaging was limited to AP radiographs, which is known to be limited in the evaluation of the complex three-dimensional anatomy of the pelvis and proximal femur. Specific to cam-type morphology, previous studies have demonstrated that evaluation of sphericity of the femoral head is assessed best using the Dunn view.^15^ We may be underreporting the prevalence of AOAA in this study cohort using only the AP pelvic images; however, it should be noted that our prevalence of AOAA is similar to other studies that used advanced imaging such as MRI and EOS.^7,11^ Finally, it should be noted that coexisting hip and spine pathologies have been reported in the literature, suggesting that patients with IS may have a predisposition to the development of hip issues.^16,17^

We observed a high prevalence of radiographic abnormalities potentially indicative of FAI in asymptomatic adolescents, further demonstrating that that the observed radiographic abnormalities are associated with a low level of specificity for FAI (not effective at ruling out the presence of hip pathology among asymptomatic hips). An LCE angle <25° or >40° was the most common abnormality, present in greater than one-quarter of the of hips. The results do not support sole reliance on radiographic abnormalities. However, radiographic abnormalities may be useful in predicting future hip pain. In our study, an abnormal Tonnis angle and acetabular retroversion were significantly associated with the development of future hip pain. These results support the judicious use of both clinical and radiographic factors in the treatment of FAI to avoid overtreatment.
